# The use of PanDrugs to prioritize anticancer drug treatments in a case of T-ALL based on individual genomic data

**DOI:** 10.1186/s12885-019-6209-9

**Published:** 2019-10-26

**Authors:** Pablo Fernández-Navarro, Pilar López-Nieva, Elena Piñeiro-Yañez, Gonzalo Carreño-Tarragona, Joaquín Martinez-López, Raúl Sánchez Pérez, Ángel Aroca, Fátima Al-Shahrour, María Ángeles Cobos-Fernández, José Fernández-Piqueras

**Affiliations:** 1 Cancer and Environmental Epidemiology Unit, National Center for Epidemiology, Carlos III Institute of Health, Madrid, 28029 Spain; 2 Consortium for Biomedical Research in Epidemiology and Public Health (CIBERESP), Madrid, 28029 Spain; 3 Department of Cellular Biology and Immunology, Severo Ochoa Molecular Biology Center (CBMSO), CSIC-Madrid Autonomous University, Madrid, 28049 Spain; 4 Institute of Health Research Jiménez Díaz Foundation, Madrid, 28040 Spain; 5 Consortium for Biomedical Research in Rare Diseases (CIBERER), Carlos III Institute of Health, Madrid, 28029 Spain; 6 Bioinformatics Unit, Structural Biology and Biocomputing Programme, Spanish National Cancer Research Center (CNIO), Madrid, 28029 Spain; 7 Hematology Department, Hospital Universitario 12 de Octubre, Madrid, 28041 Spain; 8 Department of Congenital Cardiac Surgery, Hospital Universitario La Paz, Madrid, 28046 Spain

**Keywords:** T-ALL, Next-generation sequencing technologies, PanDrugs, Precision oncology, Personalized precision medicine, Translational bioinformatics, Cancer genomics, In silico prescription, Targeted therapy, Druggable genome

## Abstract

**Background:**

Acute T-cell lymphoblastic leukaemia (T-ALL) is an aggressive disorder derived from immature thymocytes. The variability observed in clinical responses on this type of tumours to treatments, the high toxicity of current protocols and the poor prognosis of patients with relapse or refractory make it urgent to find less toxic and more effective therapies in the context of a personalized medicine of precision.

**Methods:**

Whole exome sequencing and RNAseq were performed on DNA and RNA respectively, extracted of a bone marrow sample from a patient diagnosed with tumour primary T-ALL and double negative thymocytes from thymus control samples. We used PanDrugs, a computational resource to propose pharmacological therapies based on our experimental results, including lists of variants and genes. We extend the possible therapeutic options for the patient by taking into account multiple genomic events potentially sensitive to a treatment, the context of the pathway and the pharmacological evidence already known by large-scale experiments.

**Results:**

As a proof-of-principle we used next-generation-sequencing technologies (Whole Exome Sequencing and RNA-Sequencing) in a case of diagnosed Pro-T acute lymphoblastic leukaemia. We identified 689 disease-causing mutations involving 308 genes, as well as multiple fusion transcript variants, alternative splicing, and 6652 genes with at least one principal isoform significantly deregulated. Only 12 genes, with 27 pathogenic gene variants, were among the most frequently mutated ones in this type of lymphoproliferative disorder. Among them, 5 variants detected in *CTCF, FBXW7, JAK1, NOTCH1* and *WT1* genes have not yet been reported in T-ALL pathogenesis.

**Conclusions:**

Personalized genomic medicine is a therapeutic approach involving the use of an individual’s information data to tailor drug therapy. Implementing bioinformatics platform PanDrugs enables us to propose a prioritized list of anticancer drugs as the best theoretical therapeutic candidates to treat this patient has been the goal of this article. Of note, most of the proposed drugs are not being yet considered in the clinical practice of this type of cancer opening up the approach of new treatment possibilities.

## Background

Acute leukaemia of the lymphoid lineage (ALL) is the most common form of childhood leukaemia. Based on the immunophenotype of the leukaemia cells we are able to classify ALL into T-cell acute lymphoblastic (T-ALL) and B-cell precursor (B-ALL) leukaemia. In particular, T-ALL is biologically and genetically heterogeneous with gene expression signatures that identify different biological and clinical subgroups associated with T cell arrest at different stages of thymocyte development [1], most often manifests with extensive diffuse infiltration of the bone marrow and blood involvement [2] .

T-ALL results from a multistep transformation process in which accumulating genetic alterations co-ordinately disrupt key oncogenic, tumour suppressor and developmental pathways responsible for the normal control of cell growth, proliferation, survival and differentiation during thymocyte development [1]. Despite undoubted successes, the toxicity of intensified chemotherapies treatments, chemotherapy resistance and the outcomes of patients with relapsed or refractory ALL remain poor [1, 3]. It is therefore still necessary develop appropriate strategies to enable us to identify more effective, therefore, less toxic treatments taking into account the patient genetic profile. The application of Next-Generation Sequencing (NGS) techniques has produced an unprecedented body of knowledge concerning the molecular pathogenesis of these haematological disorders allowing the discovery of multiple genetic and epigenetic alterations underpinning tumour development.

Personalized medicine is gaining recognition due to limitations with standard diagnosis and treatment [4]; due to the high rates of variability observed in clinical responses to treatments, which probably reflects underlying molecular heterogeneity. Furthermore, new classes of molecularly targeted drugs have been developed [5] although its potential could still be better utilized. Identifying which genetic variants may be targetable by current therapies presents a difficult challenge in personalized cancer medicine [6]. The question raised in this work is whether the availability of molecular data provided by whole exome and transcriptome sequencing could serve to guide the selection of site-specific treatments in a patient with T-ALL as a proof of principle. We have used the bioinformatics platform PanDrugs [7] as a feasible method to address the gap between raw genomic data and clinical usefulness, identifying genetic abnormalities that can be matched to drug therapies that may not have otherwise been considered. This could be a challenge to the implementation and uptake of genomics-based screening and diagnosis to map the appropriate actions.

## Methods

### Primary tumour and control samples

The University Hospital 12 Octubre (Madrid, Spain) provided us a tumour primary T-ALL sample (bone marrow). Tumour blasts were isolated from primary sample by flow cytometry sorting as CD7+ CD45+ cells. Sample was diagnosed as Pro-T acute lymphoblastic leukaemia according to World Health Organization Classification of Haematological Malignancies and recommendations from the European childhood lymphoma pathology panel.

Normalization next generation sequencing data is necessary to eliminate cell-specific biases prior to downstream analyses. Thymus control samples, were provide by La Paz University Hospital (Madrid, Spain). Due to Double Negative thymocytes (DN) are the less common fraction of cells multiplex these DN fractions by performing a single experiment on a pool of all DN cells, also pooling donors reduces variability. To create the initial pool of DN cells, isolation of thymocyte subpopulations were performed in five human paediatric thymuses of patients with only heart diseases aged 1 month to 4 years, removed during corrective cardiac surgery, using autoMACS Pro (Miltenyi Biotec) with appropriate MicroBeads. Immature thymocytes were enriched from thymocyte suspensions using the sheep red blood cell (SRBC) rosetting technique. Early progenitors (DN) were isolated as CD34+ cells. Purity was determined by flow cytometry using the following antibody: CD34-PE (MACS Miltenyi Biotec).

### Whole exome sequencing (WES)

DNA extraction was performed using the QIAamp DNA Mini Kit (Qiagen, Valencia, CA, USA) according to the manufacturer’s instructions. All isolated DNA samples were quantified by spectrophotometry, using NanoDrop (ThermoFisher Scientific, Waltham, MA, USA), and fluorimetry, using the Qubit® dsDNA HS and/or BR assay kits (ThermoFisher Scientific Inc.). WES analyses were performed with an Illumina HiSeq2000 sequencing platform using a paired end 2 X 100 read strategy and an Agilent’s SureSelect Target Enrichment System for 71 Mb. Sequencing will be done with a 100x of coverage. Processing of the raw data was done using RubioSeq pipeline [8] where the reads were aligned against the last version of human genome reference (GRCh38/hg38 assembly) using the BWA-Mem algorithm [9]. Alignment was then processed to (i) realign known indel regions, (ii) remove duplicate reads, and (iii) recalibrate quality scores. The variant calling process for SNVs and Indels identification was done using the combined results from GATK [10] and MuTect2 [11]. Python scripts were developed to combine variants.

### Variant annotations

Variants were annotated following the logic in PanDrugs, which integrates information from the Variant Effect Predictor of Ensembl [12] and additional databases. We used the versions 90 of Ensembl, 85 of COSMIC [13], and the releases 87.0 of KEGG [14], 1.53 of ClinVar [15], 31.0 of Pfam [16], 2018_07 of UniProt (UniProt Consortium 2018) and 69.0 of InterPro [17]. Genes included in a list with the most frequently altered genes in T-cell lymphoblastic neoplasia were also indicated.

### Massive mRNA sequencing

Total RNA was obtained using TriPure Reagent (Roche Applied Science, Indianapolis, IN, USA), following manufacturer’s instructions. RNA integrity Numbers (RIN) were in the range of 7.2–9.8. Sequencing of tumour-derived mRNA (RNA-Seq) was analysed after filtering total RNA by removal of Ribosomal RNA. Libraries were sequenced using an Illumina HiSeq2500 instrument (Illumina Inc., San Diego, CA, USA). Estimation of RNA abundance was calculated with Cufflinks2.2.1 software using the Ensembl GRCh37/hg19p5 annotation for human genome. All these molecular analyses were performed by the Sequencing and Bioinformatics services of Sistemas Genómicos S.L. (Valencia, Spain; https://www.sistemasgenomicos.com/en/) in two replicates.

### Identification of fusion transcripts and alternative splicing variants (ATEs)

Interpretation of RNA-Seq data using the predictive algorithm EricScript, a computational framework for the discovery of gene fusions in paired-end RNA-Seq data developed in R, perl and bash scripts. This software uses the BWA51 aligner to perform the mapping on the transcriptome reference and BLAT for the recalibration of the exon junction reference. In this study, we have used EricScript 0.5.5b and EnsEMBL GRCh37.73 as a transcriptome reference [18]. RNA-Sequencing data were also used to identify ATEs using CUFFLINKs [19].

### PCR, sanger sequencing

Polymerase-Chain-Reaction (PCR) and Sanger sequencing were used to validate novel mutations. Sanger DNA sequencing of PCR-amplified fusion sequences were performed with the specific primers indicated in Additional file 1: Table S1.

### PanDrugs

PanDrugs (http://www.pandrugs.org) provides a bioinformatics platform to prioritize anticancer drug treatments. The current version integrates data from 24 primary sources and supports 56,297 drug-target associations obtained from 4804 genes and 9092 unique compounds. Selected target genes can be divided into direct targets, biomarkers and pathway members [7].

During the processing PanDrugs computes a Gene Score and a Drug Score. The Gene Score (GScore, in the range of 0 to 1) measures the biological relevance of the gene and is estimated through the (i) cancer essentiality and vulnerability (by studying RNAi cell lines), (ii) relevance in cancer (using Cancer genes Census, TumorPortal, Driver Gene, OncoScope, and inclusion in a list with the most frequently altered genes in T-cell lymphoblastic neoplasia), (iii) biological Impact (using Functional impact predictors such as Variant Effect predictor from ENSEMBL 16 and different predictive algorithms, VEP relevant consequence, Essentiality score, Domains and Zygosity), (iv) frequency (GMAF 1000 genomes, COSMIC and gnomAD), and (v) clinical implications (ClinVar). The Drug Score (DScore, in the range of − 1 to 1) measures the suitability of the drug and considers (i) drug-cancer type indication, (ii) the drug clinical status, (iii) the gene-drug relationship, (iv) the number of curated databases supporting that relationship, and (v) collective gene impact.

To obtain the therapeutic options for this patient case, PanDrugs was queried 3 times with different types of molecular evidences: filtered variants, top 500 up-regulated genes and top 500 down-regulated genes. Filtered variants were provided as input for the Genomic Variants query option using a VCF file with converted GRCh37/hg19 assembly coordinates. The deregulated genes were selected using as criteria the log 2 based fold-change combined with an adjusted *p*-value < 0.05 and provided as input for the Genes query option.

In the three strategies we selected the most relevant therapies dividing them into 2 tiers: (i) tier 1 with the Best Therapeutic Candidates (therapies with DScore > 0.7 and GScore > 0.6), and (ii) tier 2 with the therapies with DScore > 0.7 and GScore > 0.5. For the filtered variants, we considered the drug-gene associations where the causal alteration matched the input variant and those without specification of causal alteration. For deregulated genes, we selected the therapeutic candidates where the alteration in the drug-gene association is an expression change or a copy number alteration (that can be translated into changes in the expression) in the same direction observed in the deregulated genes. The selected treatments in the three approaches were combined. Resistances arisen in some approach were used to exclude therapies suggested by the others.

## Results

### Clinical data evidenced a case of pro-T acute lymphoblastic leukaemia

Sixteen years old patient presented with a six weeks progressive cough, asthenia, hyporexia and lose of weight. The blood tests showed hyperleukocytosis (152 × 109/L), anaemia (99 g/L) and thrombocytopenia (83 × 109/L) with an increase of uric acid and lactate dehydrogenase (LDH). Chest X-ray presented mediastinum widening. A bone marrow biopsy was done showing 97% of blast cells with an immunophenotype compatible with a Pro-T acute lymphoblastic leukaemia. Cytogenetic analysis revealed 47, XY, + 16 [20] and 48, XY, + 9, + 16 [3] karyotypes, negative by FISH for deletion of *MYB* [6q23] and a translocation/inversion of the T cell receptor locus (TCR) (14q11).

### Molecular data revealed multiple candidate genes, fusion transcripts and alternative splicing variants

*Whole Exome Sequencing* (WES) and *Massive transcriptome sequencing* (RNA-Seq) were used to identify relevant genetic alterations including gene variants, gene expression levels, fusion transcripts and alternative splicing variants.

#### Whole exome sequencing

WES analysis and annotation process was performed as described in methods. We filtered gene variants using two main criteria: (i) population frequency, to select only somatic variants occurring in the tumour cells (GMAF or gnomAD < 0.01); (ii) functional impact of mutations, picking out those variants with high or moderate impact predicted to be pathogenic by at least two predictive algorithms. Additionally, we used the APPRIS Database to discard mutations affecting non-functional transcript-isoforms. A total of 689 gene variants, involving 308 genes, met those criteria. These genes were then categorized by GAD-Disease using the Functional Annotation tools from the Database for Annotation, Visualization and Integrated Discovery (DAVID) Bioinformatics Resources 6.8 (https://david.ncifcrf.gov/) [21]; Additional file 2: Table S2).

**Fig. 1:**
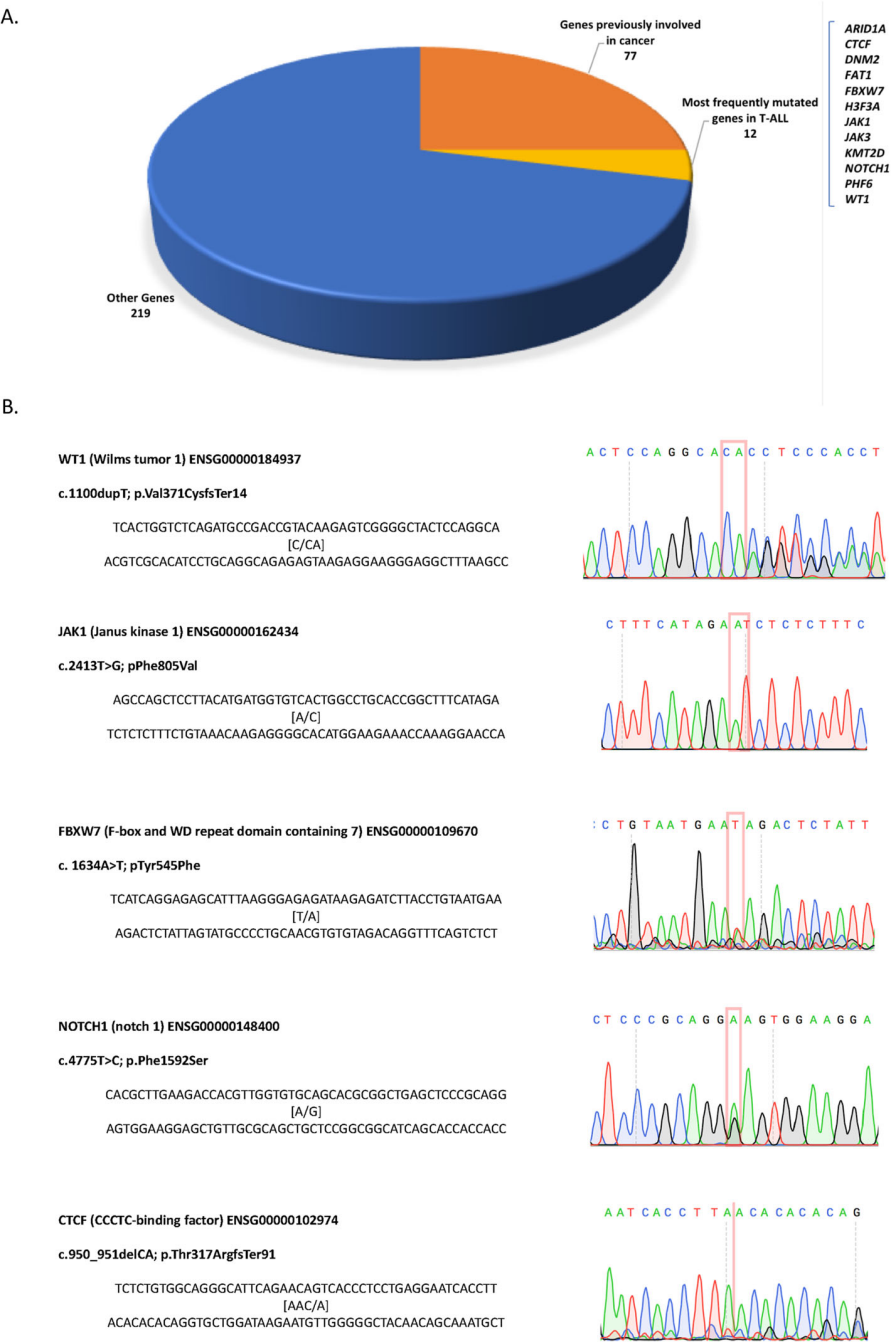
Schematic representations of the Whole Exome variants predicted to be pathogenic. **a.-** Distribution of 689 gene variants involving functional transcripts-isoforms of 308 genes, which met filtering criteria to be considered pathogenic. **b.-** Mutation validation, of fifth new gene variants detected in the patient

Scientific data available hitherto indicate that each T-ALL case only accumulates 10 to 20 biologically relevant genomic lesions, on average, as necessary events that cooperate during the development and progression of this type of leukaemia [22]. According to the information in Tumour Portal, Role Driver and Genetic Association Database (GAD_Disease data) 183 out of the 689 variants are in 77 genes previously involved in cancer. Only 12 genes with 27 presumably pathogenic gene variants were among the most frequently mutated ones in this type of leukaemia [1, 20, 23, 24]: *ARID1A, CTCF, DNM2, FAT1, FBXW7, H3F3A, JAK1, JAK3, KMT2D, NOTCH1, PHF6,* and *WT1*. Interestingly, the affectation of 4 of these genes (*DNM2, JAK1, JAK3* and *CTCF*) has been described in Early T-cell Precursor Acute lymphoblastic leukaemia (ETP T-ALL) [1, 25–27]. The T > C substitution found in the *NF1* gene is an existing variant (re2525574), which causes a stop lost effect in two defective non-functional transcripts that in addition are subjected to Non-sense Mediated Decay (NMD) (Fig. 1a).

To our knowledge 5 gene variants detected in, *CTCF, FBXW7, JAK1, NOTCH1* and *WT1* genes have not yet been demonstrated in T-ALL pathogenesis. Sanger sequencing (Fig. 1b) verified novel mutations in these genes. First, a homozygous insertion of an A after C (C to CA) in *WT1*, which generates a high-impact frameshift variant that ends in a termination codon 18 amino acids after resulting in truncation of the C-terminal zinc finger domains of this transcription factor (c.1100dupR; p.Val371CysfsTer14). Similar mutations are frequently associated with oncogenic expression of the *TLX1*, *TLX3* and *HOXA* oncogenes [28]. Second, a heterozygous presumably activating missense-variant at the pseudo kinase domain of the JAK1 protein (c.2413 T > G; pPhe805Va). Third, a heterozygous inactivating missense variant in the *FBXW7* gene (c.1634A > T; p.Tyr545Phe), which overlaps with the three main isoforms (α, β and γ). Fourth, a presumably activating heterozygous missense variant at the HD-N domain of the NOTCH protein /c.4775 T > C; p.Phe1592Ser). Fifth, an inactivating high-impact frameshift mutation at the *CTCF* gene, which generates a premature stop codon (c.950_951delCA; p.Thr317ArgfsTer91).

#### Massive transcriptome sequencing (RNA-Seq)

RNA-Seq analysis and annotation process was performed as indicated in the methods section. Significant deregulation was established calculating the log2 Fold Change (log2FC) by comparing patient sample expression data with the expression data of normal paediatric DN thymocytes (CD34+ mix), in two replicates. Absolute fold change values equal or greater than 1.5 were considered as thresholds of significance. With this stringency filtering criterium there were 6652 genes with at least one principal isoform significantly deregulated. Of these, 3575 have at least one principal isoform up regulated; 3436 exhibited at least one down regulated main isoform and, surprisingly, we detected 359 genes with at least one major isoform up and another down (Additional file 3: Table S3).

**Fig. 2:**
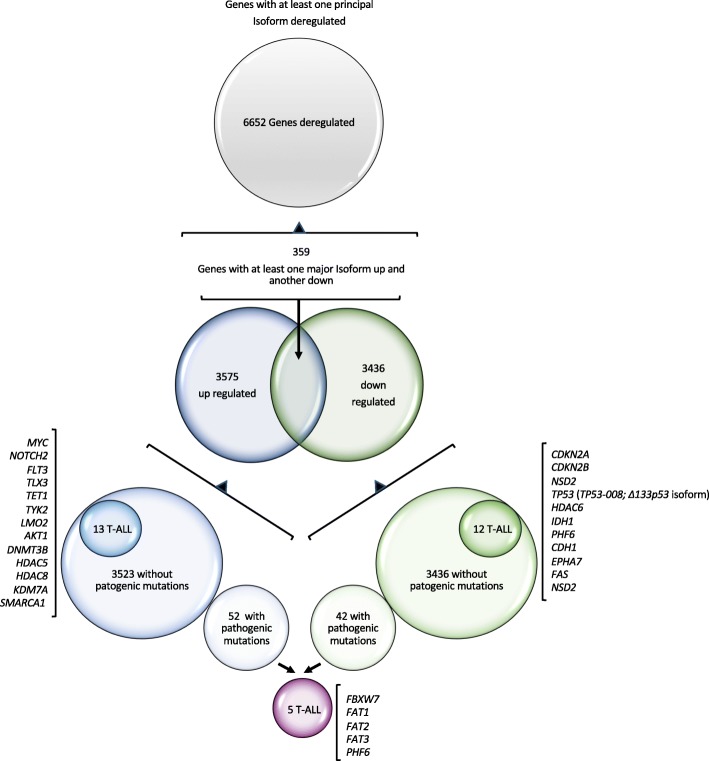
Schematic representations of significant deregulated genes.- Distribution of the 6652 deregulated genes. Significant deregulation was bases on fold changes > 1.5 (up-regulation) or < 1.5 (down-regulation) with respect to expression values in DN control samples

Cross-talk between exome and transcriptome data revealed 94 genes that exhibited pathogenic mutations and significant deregulation (52 up and 42 down) (Additional file 4: Table S4). Of them, five genes are in the list of most frequently altered ones in T-ALL (*FBXW7, FAT1, FAT2, FAT3* and *PHF6*) (Additional file 5: Table S5). Notably, 6558 genes without pathogenic mutations were significantly deregulated (3523 with some isoform up and 3393 with some isoform down) (Additional file 6: Table S6) and some of them (25 genes) are included in the list of most frequently altered genes in T-ALL (13 up and 12 down) (Additional file 7: Table S7). Up-regulated genes included *MYC, NOTCH2, FLT3, TLX3, TET1, TYK2, LMO2*, *AKT1*, *DNMT3B*, *HDAC5*, *HDAC8*, *KDM7A*, and *SMARCA1*. Down regulated genes included *CDKN2A, CDKN2B, NSD2*, *TP53* (*TP53–008; Δ133p53* isoform), *HDAC6*, *IDH1*, *PHF6*, *CDH1*, *EPHA7*, *FAS* and *NSD2* (Fig. 2)*.*

#### Fusion transcripts

Many pediatric cancers are characterized by gene fusion events that result in aberrant activity of the encoded proteins. Interpretation of RNA-Seq data using the predictive algorithm EricScript (EricScore > = 0.5) allow us to detect 126 fusion transcripts not previously described in T-ALL [20] (Additional file 8: Table S8). These fusion events identified by RNA-Seq may have unique biologic and diagnostic relevance.

#### Alternative splicing variants

Relative few significant ATEs have been reported in previous studies with T-ALL patients [20]. In our case, we detected novels junctions in *FTL3* and *KMT2D* with a known acceptor and a novel donor site that may be of functional consequences in the case of *KMT2D* gene. ATEs in *KMT2D, TCF7* and *CNOT6* might also have negative implications due to the loss of critical domains (Additional file 9:Table S9).

### Proposal of personalized and prioritized drug treatments

Identifying which genetic variants may be targetable by current therapies in this patient has been accomplished by using PanDrugs, a new computational methodology that provides a catalogue of candidate drugs and targetable genes estimated from a list of gene variants and deregulated genes provided by genomic analyses. This tool considers multiple targetable mutations, deregulations and the protein pathway-specific activity to prioritize a list of druggable genes categorized as direct targets, biomarkers and pathway members [7].

In order to evaluate the relevance of driver mutations, gene variant annotations of this patient were filtered by (i) population frequency (GMAF and gnomAD < 0.01), (ii) consequences of high and moderate impact according to Ensembl classification and (iii) affectation of canonical or unknown isoforms (Additional file 10: Table S10). An approach using the combination of the two general strategies based on gene mutations and significant gene deregulation suggested, as the best candidate selection, a total of 20 prioritized drugs supported by scores nearest to 1 in both GScore and D-Score values and should therefore be seen as the most effective approaches. All these drugs have the approval to be used in the treatment of different types of cancer (including blood cancer). Most of them would function as targeted therapy. Genes with GScore above the Tier’s threshold include mutated marker genes such as *MAP 2 K3*, *ARID1A*, *MAP4K5*, *PKHD1* and *JAK3*, which have a genetic status associated with the drug response but the protein product is not the drug target itself. Other deregulated genes, such as *NF1*, *FGFR1*, *FLT3* and *KIT*, encode proteins that can be directly targeted by a drug. Possible compensatory mechanisms of resistance and sensitivity to drugs have been taken into consideration. (Table 1).

**Table 1:**
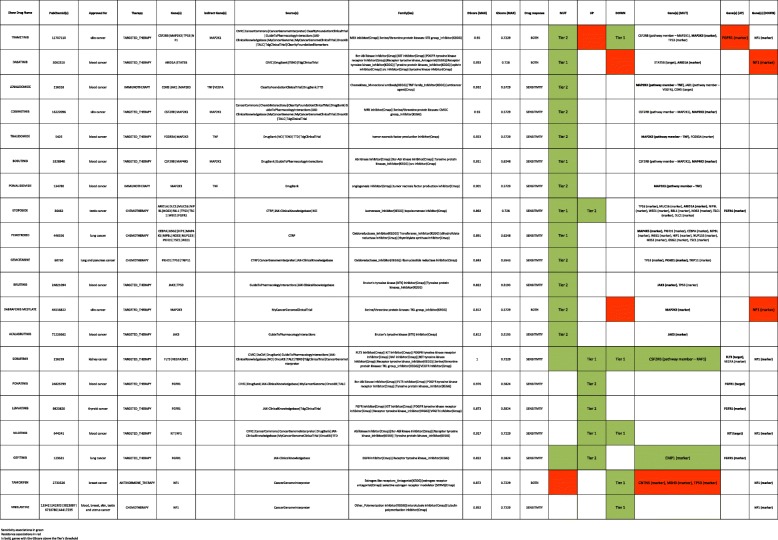
Therapeutically Proposal.- Best-Candidate therapies on the basis of genes mutated and/or deregulated (UP y genes DOWN) in which at least one of the genes linked to the drug contains the specific alteration that determines the drug-gene association

Red color indicates resistance. Green color, sensitivity. In bold, genes with the GScore above the Tier’s threshold

## Discussion

Personalized medicine to map the landscape of the cancer genome and discover new changes linked to disease is gaining recognition due to limitations with standard diagnosis and treatment. Identifying which genetic variants provided by massive sequencing analyses may be targetable by current therapies presents a difficult challenge in personalized cancer medicine. In this scenario, precision oncology requires novel resources and tools to translate the vast quantity of data generated to clinical utility [6].

The use of next generation sequencing technologies have provided an appraisal of molecular alterations that have the potential to influence therapeutic decisions involving the selection of treatment [29]. To evaluate the potential of an integrated clinical test to detect diverse classes of somatic and germline mutations relevant to T-ALL, we performed two-platform WES and transcriptome (RNA-Seq) sequencing of tumours and normal tissue. WES identifies pathogenic sequence mutations including single nucleotide variations (SNVs) and small insertion-deletions (indels); RNA-Seq detects gene fusions and outlier expression. Combined WES and RNA-Seq, is the current gold standard for precision oncology, achieved 78% sensitivity [30]. The results of our study emphasize the critical need for incorporation of NGS technologies in clinical sequencing.

For this proof-of-principle, our case study was a 16-year-old boy with an immunophenotype compatible with a Pro-T acute lymphoblastic leukaemia diagnostic. He received first-line induction chemotherapy in the conditioning regimen of the PETHEMA group; unfortunately this treatment was not effective. Allogeneic stem cell transplantation was done as a second-line therapy to treat the progression of the disease, in this case with a favorable result for the patient. Given the degree of pathogenicity of the disease, these treatments were carried out at the time in which the genetic analyzes that gave rise to this publication were being carried out. In our opinion treatment options may change is vital to improve cure rates and minimize toxicities in childhood ALL.

As indicated the PanDrugs analysis of the tumour sample for this patient identified druggable genetic alterations showing a list of 20 prioritized drugs as the best candidate selection. Since genes with GScore above the Tier’s threshold include mutated marker genes such as MAP2K3 it is not surprising that Trametinib dimethyl sulfoxide (DScore 0.95), a highly selective inhibitor of MEK1 and MEK2 activity that controls the Mitogene Activated Protein Kinase (MAPK) signalling pathway, is the first recommended option to treat this patient. This drug has proved to improve overall survival in adult patients with unresectable or metastatic melanoma with a BRAF V600 mutation [31] and could be useful for the treatment of specific T-ALL subsets [23].

Lenalidome (DScore 0.932), Thalidomide (DScore 0.923) and Pomalidomide (DScore 0.901) are immunomodulatory drugs that have shown activity against the activation of tumor necrosis factor (TNF) pathway probably through the mutation of *MAP2K3* in our patient. This means that control and effectively blocks the development of abnormal cells, prevents the growth of blood vessels within tumors and also stimulates specialized cells of the immune system to attack the abnormal cells. These drugs have been used in multiple myeloma treatment but Lenalidomide also for some myelodysplastic syndromes and mantle cell lymphoma [32].

Other antineoplastics molecular target inhibitors as Dasatinib (DScore 0.933), which inhibits STAT5B signalling [33], Bosutinib (DScore 0.921), Ponatinib (DScore 0.976) and Nilotinib (DScore 0.927) tyrosine-kinase inhibitors designed for the treatment of BCR_ABL positive neoplasms, mainly in chronic myeloid leukaemia but also acute lymphoblastic leukaemia, have also off-target effects on other tyrosine-kinases. However, Dasatinib could be discarded on the basis of criteria of resistance (shaded in red in Table 1).

In addition drugs as Ibrutinib [23] (DScore 0.822) and Acalabrutinib (DScore 0.812) Burton’s tyrosine-kinase inhibitors used in chronic lymphoid leukemia and mantle-cell lymphoma shows activity against JAK3 [34], which is mutated in our patient. Also *FLT3* [35], a gene that is upregulated in our case is inhibited by Sorafenib a kinase inhibitor drug approved for the treatment of primary kidney cancer (advanced renal cell carcinoma), advanced primary liver cancer (hepatocellular carcinoma) FLT3-ITD positive AML and radioactive iodine resistant advanced thyroid carcinoma.

Other drugs already used for T-ALL chemotherapy as Vinblastine (DScore 0.852) what causes M phase specific cell cycle arrest by disrupting microtubule assembly and proper formation of the mitotic spindle and the kinetochore or Etoposide (DScore 0.892) witch forms a ternary complex with DNA and the topoisomerase II enzyme (which aids in DNA unwinding), prevents re-ligation of the DNA strands, and by doing so causes DNA strands to break [3, 36] are also suggested by PanDrugs thus supporting the reliability of this bioinformatics application (see Additional file 11: Table S11 for further details).

## Conclusions

It is well known that complex diseases as cancer should not be considered as a single entity. Personalized medicine is a therapeutic approach involving the use of individual’s information (genetic and epigenetic) to tailor drug therapy instead of one-size-fits-all medicine. The current approach to drug development assumes that all patients with a particular condition respond similarly to a given drug. This paper provided a framework for T-ALL patients based on the use of PanDrugs to integrate whole exome sequencing and RNA-Sequencing data into the proposal of a prioritized list of drugs, which could be clinically actionable in the context of a personalized medicine of precision. This approach is toward truly precision cancer care. Furthermore drugs directed to the activity of the surrounding interactors in the biological pathway of a mutated gene could be used in combination to avoid possible compensatory mechanisms of resistance to drugs. It means that patients with different types of cancer could receive similar treatments on the basis of the genomic diagnosis. Of note, most of the proposed drugs in this T-ALL case are not being yet considered in the clinical practice of this type of cancer, opening up the approach of new treatment possibilities. At present, many of the proposed drugs are approved on the basis of clinical trials on large populations in tumours other than T-ALL so the risk of failure is lower, because the drugs have already been found to be safe, the time frame for drug reprofiling can be reduced, because most of the preclinical testing, safety assessment and formulation development will be completed. However regulatory considerations, organizational hurdles and patent considerations must be taken into account. Repurposing of these drugs for T-ALL would require validation of the results of treatments in in vitro models that have the same genetic characteristics as the samples of the patients to be treated as well as in vivo patient-derived xenografts and eventually in trials that allow repositioning of the proposed drugs.

The speed, accuracy and accessibility of next-generation sequencing (NGS) have driven the arrival of precision medicine, its mandatory to assume that this revolution must be transferred to its applicability to patients. Bioinformatics tools such as Pandrugs will allow, using the information obtained by the sequencing platforms, to improve the effectiveness of the treatments, reducing unwanted side effects and favoring survival rates.

## Supplementary information

**Additional file 1: Table S1.** Primer list. Description of primers required for Sanger sequencing.

**Additional file 2: Table S2.** WES annotation process.

**Additional file 3: Table S3.** Deregulated genes after RNA-Sequencing.

**Additional file 4: Table S4.** Results of crossing exome and transcriptome data.

**Additional file 5: Table S5.** Genes included in the most frequently altered ones in T-ALL.

**Additional file 6: Table S6.** Genes without pathogenic mutations but significantly deregulated.

**Additional file 7: Table S7.** Genes without pathogenic mutations but significantly deregulated included in the list of most frequent altered genes.

**Additional file 8: Table S8.** Fusion transcripts.

**Additional file 9: Table S9.** Alternative Splicing Variants.

**Additional file 10: Table S10.** Gene variants selected for PanDrugs.

**Additional file 11: Table S 11.** Therapies. Mutations. Deregulation UP. Deregulation DOWN.

## Data Availability

The webtool is freely accessible at http://www.pandrugs.org and through its programmatic API or docker image.

## Abbreviations

ALL: Acute leukaemia of the lymphoid lineage
ATEs: Alternative Splicing Variants
B-ALL: B-cell precursor leukemia
ClinVar: Clinical implications
DAVID: Visualization and Integrated Discovery Bioinformatics Resources
DN: Double Negative
DNA: Deoxyribonucleic acid
DScore: Drug Score
ETP T-ALL: Early T-cell Precursor Acute lymphoblastic leukaemia
GAD: Genetic Association Database
GScore: Gene Score
INDELS: Insertion-deletions
LDH: Lactate dehydrogenase
log2FC: log2 Fold Change
MAPK: Mitogene Activated Protein Kinase
NGS: Next-Generation Sequencing
NMD: Non-sense Mediated Decay
PCR: Polymerase-Chain-Reaction
RIN: RNA integrity Numbers
RNA: Ribonucleic acid
RNA-Seq: Massive transcriptome sequencing
SNV: Single Nucleotide Variations
SRBC: Sheep Red Blood Cell
T-ALL: Acute T-cell lymphoblastic leukaemia
TCR: T cell receptor
TNF: Tumor necrosis factor
WES: Whole Exome sequencing
